# Risk Assessment and Management for Potential Living Kidney Donors: The Role of “Third-Party” Commission

**DOI:** 10.3389/fpubh.2022.824048

**Published:** 2022-03-17

**Authors:** Lucia Tattoli, Davide Santovito, Ida Marina Raciti, Antonio Scarmozzino, Giancarlo Di Vella

**Affiliations:** ^1^Section of Legal Medicine, University Hospital Città della Salute e della Scienza di Torino, Turin, Italy; ^2^Clinical Risk Management Unit, University Hospital Città della Salute e della Scienza di Torino, Turin, Italy; ^3^Hospital Medical Direction, Ospedale Molinette, University Hospital Città della Salute e della Scienza di Torino, Turin, Italy; ^4^Legal Medicine Unit, Department of Public Health and Pediatric Sciences, University of Turin, Turin, Italy

**Keywords:** living organ donation, kidney transplant, informed consent, risk management, healthcare safety, “Third-Party” Commission

## Abstract

Living kidney donation is the most common type of living-donor transplant. Italian guidelines allow the living donations from emotionally related donors only after clear and voluntary consent expressed by both the donor and the recipient involved. Living donation raises ethical and legal issues because donors voluntarily undergo a surgical procedure to remove a healthy kidney in order to help another person. According to the Italian standards, the assessment of living donor-recipient pair has to be conducted by a medical “third party”, completely independent from both the patients involved and the medical team treating the recipient. Starting from the Hospital “Città della Salute e della Scienza” of Turin (Italy) experience, including 116 living kidney donations, the Authors divided the evaluation process performed by the “Third-Party” Commission into four stages, with a particular attention to the potential donor. Living donation procedures should reflect fiduciary duties that healthcare providers have toward their patients, originating from the relationship of trust between physician and patient. In addition to that, the social implications are enormous if one considers the worldwide campaigns to promote public awareness about organ donation and transplantation, and to encourage people to register their organ donation decisions. The systematic process proposed here can be a tool that proactively reduces and controls the risks of coercion, organ trafficking, vitiated consent, insufficient weighting of donative choice, that could arise especially in donors involved in living kidney donation.

## Introduction

Due to the dramatically increased number of transplant candidates listed for deceased donor transplantation over the years, in Europe patients wait on average 3–5 years for a deceased donor kidney (1, 2).

Brain-death donors are the most common source of organs, and are the core of the national organ transplant programs (3–5). The challenge is to address the discrepancy between supply and demand in organ transplantation through the expansion of the donor pool (6–8). The main strategies include the use of living donors, the promotion of donation after brain death but also of donation after circulatory death (1, 3, 7, 9, 10).

Living kidney donation is the most common type of living-donor transplant (2, 11). The data collected by the Italian National Transplant Center revealed an increase of kidney transplants from living organ donations (LOD) in recent years, although the number of living donor transplants is still low compared to the number of patients on the waiting list (12).

Several factors account for LOD increase (1, 7, 8, 10, 11, 13–15):

- the effectiveness of the organ procurement system,
- the progress in transplant surgery,
- the better graft and patient survival when compared with cadaver donor transplantation,
- the surgery planned in advance when a living donor is available with reduction or prevention of the need for dialysis,
- the growing need for organs,
- the shortage of available dead donor organs.

There are different types of living donation for kidney transplantation (7, 8, 11, 16):

- directed donation: genetically-related donation (person who is a blood relative of the potential recipient), emotionally-related donation (there is relationship between donor and recipient, e.g., spouse, partner, and friend); paired-donation (an emotionally related donor is incompatible with the potential recipient and they are matched with another donor and recipient in a similar situation); pooled donation (the pair is matched with other donors and recipients from a pool of pairs in similar situations);
- non-directed altruistic donation or good Samaritan donation: the donor does not have any relationship with the recipient.

Italian guidelines allow the living donations from emotionally related donors only after explicit and voluntary consent to the donation expressed by the donor and recipient involved (17).

So, in addition to the clinical and immunological assessments and a psychological exam, living donors are also assessed regarding their reasons for donating, their understanding of the potential risks and of the real likelihood of the transplant being successful in terms of graft and patient survival, the bond of affection between the donor and recipient, and the honesty of their free and informed consent (17–20).

## Living Organ Donation

### Epidemiological Data

In 2020, 129,681 solid organs were transplanted annually, with a 17.6% decrease over 2019 (in 2019, a 4.8% increase over 2018 have occurred). Among 80,926 kidney transplants performed worldwide, 31.07% were from live donors (21).

In 2020, the Covid-19 pandemic led to an overall decrease in the Italian transplant activity of <10%, compared to 2019. A decrease of −19.4% in transplants from living donors was registered compared to 2019. As a result, in 2020 patients waited for 2.07 years on average for a deceased donor kidney (12). The reduction in the number of living donor transplants was due to the suspension of programmable and non-urgent surgical activities adopted by several hospitals, because of the reorganization and the increase of intensive care places during the health emergency (12, 22).

In Italy, the majority of kidney transplants in 2020 (*n* = 169) were performed by A.O.U. Città della Salute e della Scienza of Turin, followed by A.O. of Padua (*n* = 139) and Ospedale Civile Maggiore of Verona (*n* = 96) (12).

### Living Organ Donation Regulation

The Member States of the European Union are active in the field of living organ donation starting from the Directive 2010/53/EU, also known as The European Organs Directive (Directive 2010/53/EU of the European Parliament and of the Council of July 7, 2010 on Standards of Quality and Safety of Human Organs Intended for Transplantation) stating that the handling and disposal of human organs should respect the fundamental rights and the human body (23). Thus, it must conform to the Oviedo Convention on Human Rights and Biomedicine and the Additional Protocol on the transplantation of organs and tissues of human origin (2, 8, 11, 24, 25).

The evaluation, of the donor in particular, must comply with a rigorous process. In 2004, the Consensus Statement of the Amsterdam Forum on the Care of the Live Kidney Donor developed an international standard of care with a position statement of the Transplantation Society regarding the responsibility of the community for the live kidney donor (26). It was stated that the potential donor: (i) must receive a complete medical and psychosocial evaluation; (ii) must receive complete information of the evaluation process, the results of testing, the risks of donor nephrectomy, the expected transplant outcomes, the alternative renal replacement therapies; (iii) should understand the information in the consent process. The donor must voluntarily decide to donate, and he/she has the right to withdraw from organ donation at any time during the procedure for any reason.

In Italy, Law n. 91 dated 1st of April 1999 introduced the National Transplant Center (CNT) as the technical body of the Ministry of Health (https://www.iss.it/web/iss-en/transplants). The Italian CNT supports the correct information on the issues of donation and transplantation through the promotion of information, education and cultural growth of the general public especially in the field of living transplantation.

In 2010 CNT produced the main informative document about the living kidney donation program (“*Documento informativo sul programma di trapianto di rene da donatore vivente*”, https://www.salute.gov.it/imgs/C_17_pubblicazioni_1186_allegato.pdf (27). The following principles were stated:

- the donor must receive adequate information on the type, extent and likelihood of risks, on possible alternatives for the recipient;
- the donor must not be subjected to pressure, coercion, solicitation, economic or any other type of incentive;
- the assessment of the eligibility to be a donor must be carried out by a medical team that is completely independent from both the patients involved and the professionals who follow the transplant process;
- the donor's right to withdraw consent until the last moment before surgery must be guaranteed;
- the donation must not be a source of profit or be commercialized;
- the donor must undergo rigorous medical examinations aimed at identifying any physical or psychological contraindications;
- long-term care must be ensured for donors, as well as recipients.

Law no. 24 (March 8, 2017) focuses on the improvements of the quality and safety of healthcare as key elements in the preservation of the constitutionally protected right to enjoy good health (28, 29).

## Risk Assessment in Living-Organ Donation

Living donation raises ethical and legal issues because donating an organ exposes a healthy person to the risk of and recovery from unnecessary major surgery, with a permanent injury of psycho-physical integrity (4, 30).

The organs and tissues donation system is a complex process. In living donation, the most important issue is a free and informed consent correctly obtained from the donor and the recipient. The donors must be aware of their donation act and must express their will in serenity and without any coercion, considering that the donation cannot and must not represent any opportunity for profit (15, 31, 32). If informed consent and the absence of profit are the two cornerstones that should improve the living donation, the risks are an invalid or vitiated consent, or even worse, a consent affected by interests totally unrelated to solidarity, foundation of organ's donation (16, 24, 32).

This is well summarized in the definition of organ donation provided by the European Parliamentary Research Service, as “the act of giving one or more organs (or parts thereof), without compensation, for transplantation into someone else” (25).

It is therefore clear that in the context of living kidney transplantation it is more than ever mandatory to protect the donor's health and safety and to demonstrate to the community the effectiveness and transparency of the service during the whole process.

The procedure should be documentable and transparent in the light of a free and informed consent, given by a healthy subject, without coercion and/or disinformation, and in the absence of any interest and/or utility. Healthcare safety is also based on these principles (33).

The article 1 of Law 24/2017 stated that safety is guaranteed through proper prevention tools and healthcare risk management, in connection with the most effective use of structural, technological and organizational resources available (28, 29).

In living donation, the risks for the donor and recipient can come from missed surveillance as imposed by the guidelines. This could affect patients' trust in healthcare professionals and transplantation Organizations concerning living donation.

The biggest risks, regarding the safety of the living donation process, affect the donor, as resumed in the following (8, 11, 16, 30, 31, 34):

- the consent is absent;
- the consent is not informed or not fully informed;
- the consent is coerced or secondary to practices such as organ trafficking or commercialization;

the consent is invalid (the individual must have the legal capacity to do so and the cognitive ability to understand the nature and consequences of the procedure), the donor is not genetically, legally or emotionally related to the recipient.

In addition to the clinical and immunological assessments, the living donors have to be assessed with regard to their reasons for donating, their understanding of information provided by physicians, the nature of the relationship between the donor and recipient, and the validity of the consent.

For this purpose, according to national guidelines on kidney transplantation from living donors, donors and recipients are assessed by a medical “Third-Party” Commission (TPC), independent from the transplantation team. The TPC was instituted by the Italian National Transplant Center (NTC) in living-donor kidney transplantation guidelines released in 2002 (15, 17).

The Decree n. 116/2010 by the Minister of Health, providing the regulation of living organ (kidney and liver) donor transplantation, underlined the dual role of TPC of verifying and monitoring the process. This evaluation takes place at the last stage of the donation process. Only after the successful conclusion of this process, the transplant can occur.

With particular attention to the donor as the most exposed person to the risks, it is requested:

1) the verification of informed, free and conscious consent through verbal and written information, and of the relationship between donor and recipient (if genetically, legally or emotionally related);
2) the control of the procedure to prevent risks of organ trafficking or commercialization or coercion.

The tools of TPC are:

- medical records, identity documents (objective assessment);
- structured interview with the potential donors.

At last, TPC expresses a joint opinion.

The evaluation process performed by TPC can be divided into four stages: 1° or *analytical stage*; 2° or *listening stage;* 3° stage *of the legal requirements;* 4° *stage of traceability*.

## The Experience of the Hospital “Città Della Salute e Della Scienza” of Turin, Italy

According to the national guidelines, in 2010 the Hospital “Città della Salute e della Scienza” of Turin, Italy, established a TPC for kidney transplantation, including a forensic pathologist, a nephrologist and a medical director, all independent from the transplantation team.

Between April 2011 and December 2020, TPC assessed 116 potential donor-recipient pairs, examining their medical records, and interviewed potential donors and recipients, recording on evaluation reports and eligibility determination forms. Donors and recipients were assessed separately; the Commissioners, then, expressed a joint opinion.

The mean time between identifying a living donor and donation is 6–12 months.

The data are described in aggregate form. In total, 232 subjects were examined (Table 1), consisting of 78% females and 22% males among donors, and 27% female and 85% males among recipients. The donors' average age was 54.75 years. The recipients' average age was 43.51 years. The most representative age group was 51–60 years old for donors, and 41–50 years for recipients.

**Table 1:** Summary of the results from potential donor-recipient pairs' assessment by the “Third-Party” Commission (Hospital “Città della Salute e della Scienza” of Turin, Italy).

	***N%***	**Age average**	**Age group**		**Genetically-** **related donation** **58%**	**Parents 63%** **Brothers/sisters 34%** **Other relatives 3%**
Donors	78% F 22% M	54.75 yrs	51–60 yrs	Relationship D/R	Non-genetically- related donation 42%	Married couples 75% Unmarried couples 10% Other 15%
Recipients	27% F 85% M	43.51 yrs	41–50 yrs			

The relationship between donor and recipient included genetically-related donation (58%) and non-genetically-related donation (42%). Among the genetically-related donation, parents were 63% (mostly the mothers), brothers/sisters were 34%, and other relatives 3%. Among non-genetically-related donations, married couples were 75%, unmarried couples 10% and other 15% (relatives-in-law, coworkers, friends).

The recipients' kidney pathologies, for which the living-donor transplant was proposed, included, for the most cases, acquired glomerular/tubular/interstitial nephropathy and congenital cystic nephropathy.

The donation was prompted by the donor in 80% of cases; the living donation was suggested by the physicians of the recipient in most cases.

For most donors, the decision arose from the desire to help a loved one to improve their quality of life and to avoid dialysis, which was perceived as a very stressful family situation. Several donors made their decision after rationally considering the pros and cons for the recipient's life in the absence of alternatives for relief. Those donors were unable to accept the recipient's risks deriving from cadaver transplantation and a too long waiting list. In some cases, the reason for the donation was to improve the familiar setting which was severely affected by the recipient's pathology. Thus, an active gesture “had” to be made, especially if alternative donor options were lacking. On the other hand, the recipients' reactions were happiness with no attempt to influence the donor's decision, ranging to the concern for the consequences for the donors with initial opposition to the donor's decision. In no cases were the donors' and recipients' consents withdrawn, and no pressures or obligation from the outside were mentioned by the pairs or perceived by the Commission.

## Discussion

Living-donor transplantation represents an alternative—not a substitution—to the waiting list for a deceased donor organ. Nevertheless, living transplant often is the only treatment option to improve or save the life of patients with an end-stage kidney failure (18). It is well known that living-donor organ transplants are associated with fewer complications than cadaver transplants, a better outcome, and a longer survival time of the donor organ (7, 17, 18).

Living organ donors are a unique group of patients because they voluntarily undergo a surgical procedure to remove a perfectly healthy kidney to help another person. Their evaluation has to take into account all the physical, psychological, and social risks to the individual donor (35, 36). Also, the public trust of the healthcare community and the health system should not be compromised.

In Italy, the donation from living people is allowed notwithstanding the Art.5 of Civil Code, which *prohibits any actions on one's own body when causing a permanent damage to physical integrity or when violating Law, public order or decency*. In fact, the Law n. 458 of June 26, 1967, allows the living donor kidney transplantation. The regulatory framework of living donation includes the Italian Code of Medical Deontology (last edition in 2014) where the article 39 states that *living organ and tissue donations allowed only for diagnostic, therapeutic, or scientific research purposes and if it does not cause permanent damages to donor's physical or psychic integrity, according to the Law. The donation must be a no-profit and no-commercial activity, a written, free and informed consent must be given by the donor*.

The inspiring principles are the same of those underlined in the Amsterdam Forum (26).

The selection and the assessment of donor eligibility must follow appropriate standards. These include a specific and informed consent process which should make the potential donors aware of the risks of their upcoming procedure, as well as future prospects of a life with only one kidney. Consent may be withdrawn at any time before the transplant, and this should be clearly written and explained (8, 25, 31, 34).

The importance of the evaluation of motivations, in addition to the clinical, immunological and psychological assessments, arises from the peculiar nature of unrelated living donors in kidney transplantation, which is much debated. The idea behind the PTC is the creation of an independent committee that assesses the donor's motivation and the understanding of the process, and that confirms lack of coercion or commercial activity, even if this proves difficult at times.

Starting from the experience of the Hospital “Città della Salute e della Scienza” of Turin, Italy, in living kidney donation, the Authors divided the evaluation process performed by TPC into four stages, with a particular attention to the potential donor who is the one most exposed to ethical risks.

### 1° or Analytical Stage

Evaluation of the medical records (especially the signed informed consents to the transplantation surgery) with regard to the donor's age, the relationship with the recipient, risks and potential consequences of the donation (medical, psychological, social, professional, financial, insurance limitation, quality of life for both the donor and for the recipient). The previous psychological assessments are evaluated to better understand the reasons for donation, the pair's emotional relationship, the support of the pairs' family, the expectations regarding the health of the couple and to understand if the potential donor is mentally fit for the procedure. Any coercion, unethical or financial practices between donor and recipient could be ruled out at this point.

### 2° or Listening Stage

Structured interviews are carried out with the donor and recipient, regarding the whole donation's process, and the information received by the donor about his/her health, the surgical risks, and the potential consequences of the donation. The consistency of donor and recipient's interviews is assessed.

### 3° Stage of the Legal Requirements

The final donor's consent must be free and informed, and both donor and recipient have the option to withdraw at any time from the donation process. The Commission definitely excludes any coercion and opportunity of profit and/or other utility from donation, evaluates the impartiality of the records and the transparency of the process. TPC expresses a written joint opinion, and the outcome can be positive or negative. In the latter case, the Commission could suggest supplementary assessments or more time to make the decision filling any gap in the informed consent process.

### 4° Stage of Traceability

It includes the recording of the eligibility determination forms of the potential donor and recipient, and the conclusive evaluation report which all Commissioners must sign. A copy of the conclusive evaluation is provided to the donor/recipient pair and exhibited to the judicial authority that ultimately authorizes the donation.

This systematic process of the TPC proposed here could be a tool that proactively reduces and controls the risks of coercion, organ commercialization, lack of information, insufficient weighting of donative choice, that can arise in living kidney donation.

All living donation procedures should reflect fiduciary obligations that healthcare providers have toward their patients, deriving from the relationship of trust between physician and patient. The social implications are also wide if one considers the worldwide campaigns to promote public awareness about organ donation and transplantation, and to encourage people to register their organ donation decision.

## Data Availability Statement

The raw data supporting the conclusions of this article will be made available by the authors, without undue reservation.

## Author Contributions

LT and DS conceived and designed the study. LT, DS, GD, IR, and AS contributed to the writing of the manuscript. GD made the critical revision. All authors contributed to the article and approved the submitted version.

## Conflict of Interest

The authors declare that the research was conducted in the absence of any commercial or financial relationships that could be construed as a potential conflict of interest. The handling Editor DF declared a past co-authorship with one of the author LT at the time of review.

## Publisher's Note

All claims expressed in this article are solely those of the authors and do not necessarily represent those of their affiliated organizations, or those of the publisher, the editors and the reviewers. Any product that may be evaluated in this article, or claim that may be made by its manufacturer, is not guaranteed or endorsed by the publisher.
